# Ultrasound field characterization and bioeffects in multiwell culture plates

**DOI:** 10.1186/s40349-015-0028-5

**Published:** 2015-06-30

**Authors:** Upen S Patel, Sleiman R Ghorayeb, Yuki Yamashita, Folorunsho Atanda, A Damien Walmsley, Ben A Scheven

**Affiliations:** School of Dentistry, College of Medical and Dental Sciences, University of Birmingham, St Chad’s Queensway, Birmingham, B4 6NN UK; School of Engineering and Applied Sciences, Ultrasound Research Laboratory, Hofstra University, Hempstead, NY USA; Immunology and Inflammation—FIMR, North Shore Hospital, Manhasset, NY USA

**Keywords:** Ultrasound, Long-wave, Multiwell, Murine, Dentin, Pulp, Repair, Regeneration

## Abstract

**Background:**

Ultrasound with frequencies in the kilohertz range has been demonstrated to promote biological effects and has been suggested as a non-invasive tool for tissue healing and repair. However, many challenges exist to characterize and develop kilohertz ultrasound for therapy. In particular there is a limited evidence-based guidance and standard procedure in the literature concerning the methodology of exposing biological cells to ultrasound *in vitro*.

**Methods:**

This study characterized a 45-kHz low-frequency ultrasound at three different preset intensity levels (10, 25, and 75 mW/cm^2^) and compared this with the thermal and biological effects seen in a 6-well culture setup using murine odontoblast-like cells (MDPC-23). Ultrasound was produced from a commercially available ultrasound-therapy system, and measurements were recorded using a needle hydrophone in a water tank. The transducer was displaced horizontally and vertically from the hydrophone to plot the lateral spread of ultrasound energy. Calculations were performed using Fourier transform and average intensity plotted against distance from the transducer. During ultrasound treatment, cell cultures were directly exposed to ultrasound by submerging the ultrasound transducer into the culture media. Four groups of cell culture samples were treated with ultrasound. Three with ultrasound at an intensity level of 10, 25, and 75 mW/cm^2^, respectively, and the final group underwent a sham treatment with no ultrasound. Cell proliferation and viability were analyzed from each group 8 days after three ultrasound treatments, each separated by 48 h.

**Results:**

The ultrasonic output demonstrated considerable lateral spread of the ultrasound field from the exposed well toward the adjacent culture wells in the multiwell culture plate; this correlated well with the dose-dependent increase in the number of cultured cells where significant biological effects were also seen in adjacent untreated wells. Significant thermal variations were not detected in adjacent untreated wells.

**Conclusions:**

This study highlights the pitfalls of using multiwell plates when investigating the biological effect of kilohertz low-frequency ultrasound on adherent cell cultures.

## Background

Investigating the therapeutic use of ultrasound to promote biological tissue healing and repair poses many challenges to the researcher when studying the effect on cells *in vitro*. Ultrasound propagation occurs via the transfer of energy from particle to particle [1]. This results in areas of compression and rarefaction, and it is the effect of this mechanical movement on cells that is studied. The ultrasound field is not homogenous and is prone to reflection and attenuation when the field encounters a boundary between different media [2]. The challenge is to control and reproduce the parameters of the ultrasound wave that affect the cells in *in vitro* culture. There is limited evidence-based guidance in the literature concerning the methodology of exposing biological cells to ultrasound *in vitro*. A study by Hensel et al. [3] investigated megahertz ultrasound-wave propagation characteristics in four commonly used setups to expose ultrasound to cells in culture wells; well on transducer, well on water surface, sealed well, transducer in well. Their results indicated that all four of these approaches produced some degree of variability due to reflecting surfaces. A setup with no liquid-air interface would provide the most reproducible, and hence transferable, results. The authors recommended that a culture well be devoid of air and water-proof sealed such that it could be submerged within a water tank. Ultrasound would then be generated at a distance, ensuring the most homogenous portion of the ultrasound field (far-field) would be exposed to the cells. The authors of the study considered a single well setup, however, there are many studies in the literature where multiwell plates have been used to study the effects of ultrasound on cell culture [4–17]. Recommendations from Hensel et al. [3] may be applicable to multiwell plates but it is important to consider divergence of the ultrasound field and its scope of interaction with adjacent wells within the same multiwell plate.

The majority of studies that investigated the therapeutic effects of ultrasound on biological cells use pulsed ultrasound with a frequency in the megahertz range [18–20]. However, there are a number of studies that demonstrate biological effects with the use of ultrasound with a frequency in the kilohertz range [5, 7–9, 11, 13, 21–24]. Ultrasound in the kilohertz frequency range has a longer wavelength compared to megahertz ultrasound. This characteristic allows for greater penetration through living tissue or dense tissue, such as dental enamel or bone, making it potentially more effective than megahertz ultrasound [25, 26]. Therefore, low-frequency ultrasound may ideally be suited for therapeutic applications involving deep sites of injury or dense hard tissues, such as bone and tooth repair [24].

The nature of ultrasound beam propagation, from its source, to the cells, and further, causes the culture plastic, on which the cells are grown, to both attenuate and reflect the ultrasound wave. The degree of attenuation will vary by method of exposure, as described by Hensel et al. [3] and the manufacturer design of a multiwell plate. The energy absorbed by a multiwell plate during the ultrasound treatment of cells in a specific culture well has the potential to inadvertently affect cells cultured in the other wells of the of the same plate. Fung et al. [27] reported that an ultrasound field with a frequency of 1.5 MHz is well-delineated and generally linear. However, an ultrasound field with a frequency in the kilohertz range is considered to be diffuse. This characteristic of low-frequency ultrasound implies that it could affect adjacent wells in a multiwell plate when used in *in vitro* studies. It can be postulated that the attenuated ultrasound energy results in heating of the multiwell culture plate or resonance causing vibrations in each of the wells in the plate. Investigation of a biological effect in an adjacent culture well without a thermal change will add to the debate of a thermal and non-thermal mechanism of an ultrasound induced biological effect [28–32].

This study aims to characterize a low-frequency ultrasound field to investigate its propagation and divergence. We have previously studied the effects of ultrasound on dental cells with an odontoblast-like cell line, MDPC-23 [8, 9, 23, 33]. A similar model will be used; however, the treatment of these cells with ultrasound will be modified to investigate the effects on (non-treated) cells cultured in adjacent wells of multiwell plates. A spatial beam plot will identify the risks to adjacent wells when a multiwell plate is used for experiments involving *in vitro* cell culture.

## Methods

Ultrasound was generated at a frequency of 45 kHz (DuoSon, SRA Developments Ltd, Ashburton, UK). The system was preprogrammed by the manufacturer to provide three modes of continuous ultrasonic output at spatial-average intensities of 10, 25, and 75 mW/cm^2^ and calibrated using a radiation force balance (SRA Developments Ltd, Ashburton, UK). The DuoSon single-element transducer is unfocused and has an effective radiating area of 16.3 cm^2^ when generating ultrasound at a frequency of 45 kHz.

### Experimental setup for ultrasound-field characterization

**Fig. 1:**
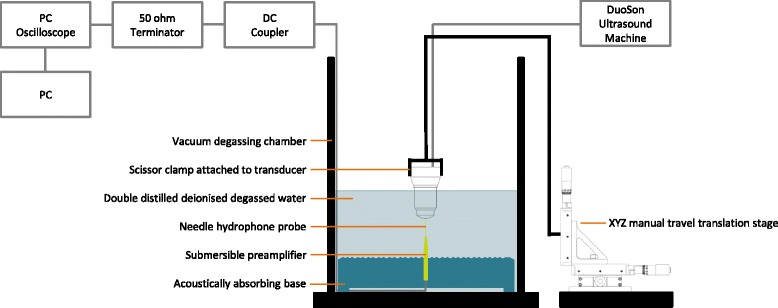
Experimental setup for ultrasound-field characterization. Annotated diagram describing the setup of equipment for measuring the ultrasound field generated from the DuoSon ultrasound machine

A vacuum degassing chamber was constructed from plastic (Applied Vacuum Engineering, Bristol, UK) with a curved internal surface to reduce ultrasonic reflections. An acoustically absorbing base was constructed of a combination of rubber and Apltile SF5048 (Precision Acoustics, Dorchester, UK). A 1.0-mm needle hydrophone probe (Model 1452; Precision Acoustics, Dorchester, UK) connected to a HP Series Submersible Preamplifier (PA09022, Precision Acoustics, Dorchester, UK) was held in place vertically by the Apltile SF5048 material. The chamber was filled with 12 L of double distilled deionized water and air evacuated to achieve a vacuum. The water was degassed for 12 h with a vacuum of 0.95 bar. The DuoSon transducer was positioned vertically in line over the hydrophone, with their central axes aligned, and its movement was controlled by an XYZ manual travel translation stage (Thorlabs Inc., Newton, NJ, USA) as shown in Fig. 1. Both the transducer and needle hydrophone probe were submerged for 4 h. This mimicked the conditions present when the hydrophone was calibrated. Voltage measurements were recorded using a PC oscilloscope (PicoScope 5203; Pico Technology, St Neots, UK). The hydrophone and preamplifier were connected to a DC Coupler (DCPS038; Precision Acoustics, Dorchester, UK), and the signal was passed through a 50-Ω Terminator (TA051 Feed-Through Terminator; Pico Technology, St Neots, UK) prior to connecting to the PC oscilloscope (Fig. 1). The transducer face was positioned 50 mm below water level, and maximum voltage measurements and frequency were recorded at ten vertical points from the transducer at 1-mm intervals from the transducer face. The transducer was displaced horizontally and ten vertical measurements were taken at a further five positions from the transducer face at 5-mm intervals.

### Ultrasound-field calculation

These measurements were recorded for all three of the preset and pre-calibrated ultrasound intensities (10, 25, and 75 mW/cm^2^). The maximum voltage and frequency measurements were used to calculate the average ultrasound intensity at each horizontal position from the transducer as described previously [26]. Initially, the pressure value was calculated using Eq. 1.

1 $$ p=\frac{V}{K} $$

where *p* is the acoustic pressure, *V* is the maximum voltage measured, and *K* is the calibration factor (certificate: U3105, calibration carried out by National Physics Laboratory, London, UK). The needle hydrophone was calibrated over a frequency range of 10–100 kHz at 5-kHz intervals. Interpolation was used to determine the equivalent calibration factor based on the frequency recorded during the measurement. Subsequently, the acoustic intensity (*I*) was calculated using Eq. 2.

2 $$ I=\frac{1}{T_{prf}}\kern0.24em {\displaystyle \int}\frac{p^2(t)}{\rho \kern0.24em c}\kern0.24em dt $$

where *T*_*prf*_ is the pulse-repetition period, *ρ* is the density of the propagating medium, and *c* is the velocity of sound in the same medium (1480 m/s). Hydrophone sensitivity is rarely constant as a function of frequency, and interpolation to determine the correct calibration factor may cause erroneous results. Full-waveform deconvolution was employed, and Eq. 1 was modified to utilize Fourier transformation. This is shown in Eq. 3.

3 $$ {\mathrm{\Im}}^{-1}\left\{\frac{\mathrm{\Im}\left(V(t)\right)}{K(f)}\right\}=p(t) $$

Intensity was again derived using the acoustic pressure calculated using Eq. 3. Both intensity values were plotted against distance from the long axis of the hydrophone.

### Ultrasound treatment apparatus setup

**Fig. 2:**
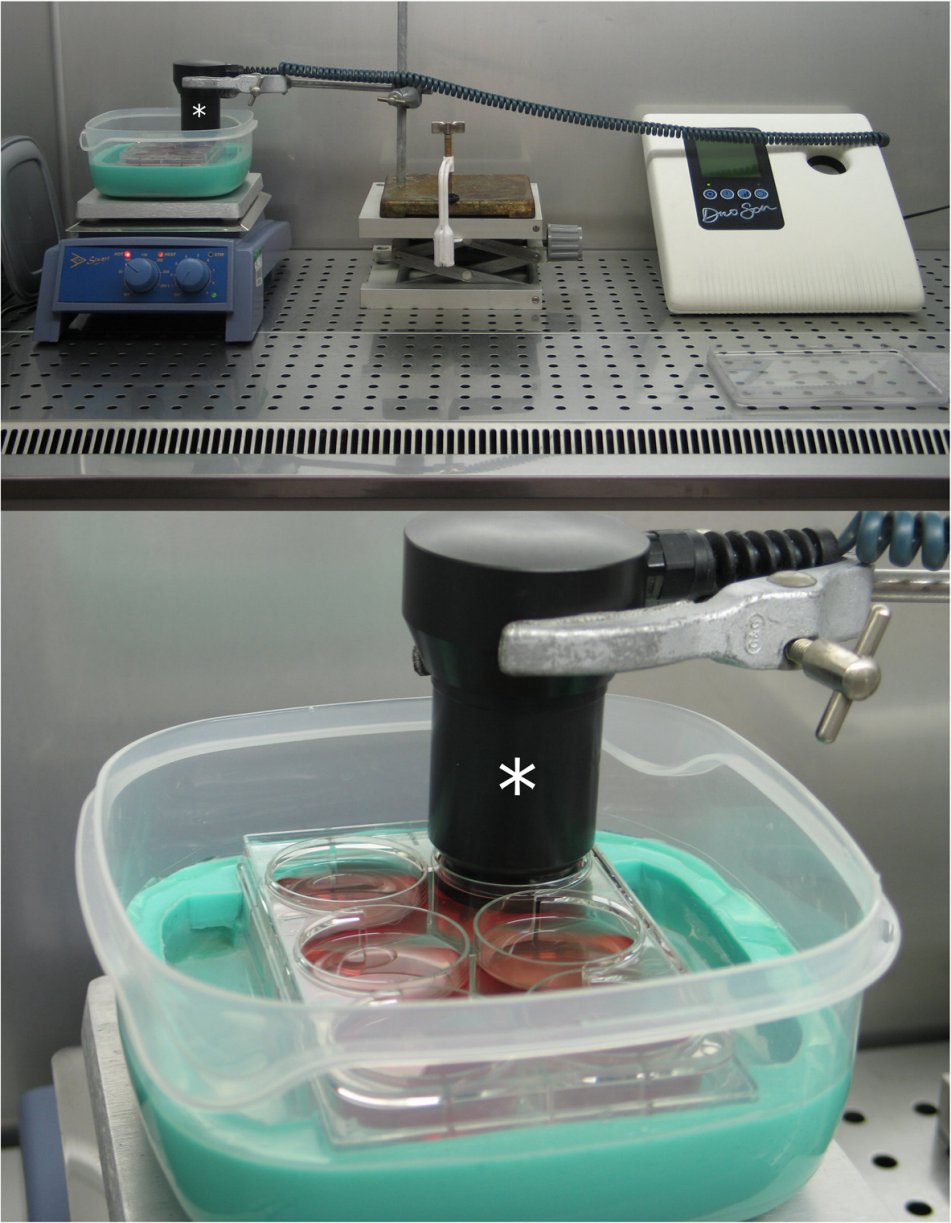
Treating biological cells with ultrasound in multiwell culture plates. DuoSon with transducer (identified by *) clamped in position in a laminar flow hood (*top*) and a close-up of a six-well plate supported by silicone in a water bath with the transducer submerged in culture media (*bottom*)

**Fig. 3:**
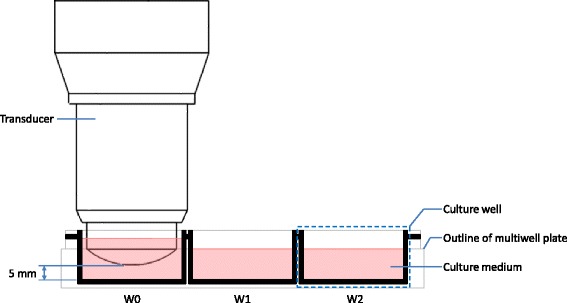
Transducer positioning in a multiwell culture plate. Annotated diagram describing the position of the transducer face from the base of the culture well. The transducer is only inserted in the culture medium of the W0 culture well of each multiwell plate

A six-well culture plate (Costar® tissue-culture treated; Corning®, Tewksbury, MA, USA) was supported in a water bath by silicone rubber (Fig. 2) to minimize reflections [8, 9]. The water bath was placed on a thermostat-controlled hot plate to keep the culture medium in each well of the six-well plate at 37 °C. The entire setup was placed in a laminar flow hood together with the DuoSon to prevent infection (Fig. 2). The transducer was clamped to a scissor stand to allow for straightforward insertion and removal from the culture well. The transducer face was positioned 5 mm from the culture surface in each culture well (Fig. 3). The thickness of the culture plastic at the base of the culture well is 1.27 mm.

### Cell number and viability

An immortalized mouse cell line of odontoblast-like dental pulp cells, MDPC-23 [9, 26, 33, 34], were cultured with Dulbecco’s Modified Eagle Medium (DMEM high glucose; Biosera, UK) supplemented with 10 % fetal bovine serum (Biosera, UK), 1 % penicillin/streptomycin (Sigma-Aldrich®, UK), and 200 mM glutamine (GlutaMAX™; Gibco®, Invitrogen™, UK) in a humidified incubator with 5 % carbon dioxide in air at 37 °C. 50,000 cells (day 0) were seeded in each of the three wells in one row of twelve six-well plates. These cells subsequently formed an adherent monolayer. The medium was replenished on days 1, 3, 5, and 7 with ultrasound treatment on days 2, 4, and 6. The DuoSon transducer was submerged into the culture medium of the first well (W0) at the corner of each plate for 5 min. Ultrasound treatments were carried out in triplicate and included a sham treatment where the transducer was submerged into the culture medium (Figs. 2 and 3) for the same length of time without the DuoSon producing an ultrasonic output. Prior to submerging the transducer into the culture medium, the transducer was wiped with 70 % alcohol and washed with sterile culture medium. Only the W0 well in each six-well plate was treated with ultrasound at a frequency of 45 kHz and each plate treated with a different intensity; no power (sham), 10, 25, and 75 mW/cm^2^. Each well was treated for 5 min. On day 8 of culture, cells were detached using a 2.5 g/l Trypsin in 0.2 g/l EDTA solution (Sigma-Aldrich®, UK) from the W0 well. Cells from the adjacent (W1) and distant (W2) wells in each six-well plate were collected using the same method. Cell counts and viability were measured with trypan blue (Sigma-Aldrich®, UK) staining and a Neubaeur haemocytometer (Neubaeur, Frankfurt, Germany).

### Temperature measurements

The apparatus was set up as described earlier, and a six-well plate, containing 9 ml of culture medium in each well, was taken from an incubator at 37 °C and positioned in the water bath. A thermocouple (TC-PVC-T-24-180; Omega Engineering Limited, Manchester, UK) was used to measure the temperature rise of the culture medium in the well. Temperature measurements were also taken adjacent and distant to the culture well where the transducer was submerged. The thermocouple was positioned on the culture plastic, at the center of each well. A measurement was taken every 30 sec to ensure variations in temperature over the maximum treatment time of 30 min while ultrasound was produced by the DuoSon at the three 45-kHz ultrasound intensities; 10, 25, and 75 mW/cm^2^. Measurements were taken every 30 sec to ensure specific time points would be recorded to ascertain treatment times.

## Results

### Ultrasound beam characterization

**Fig. 4:**
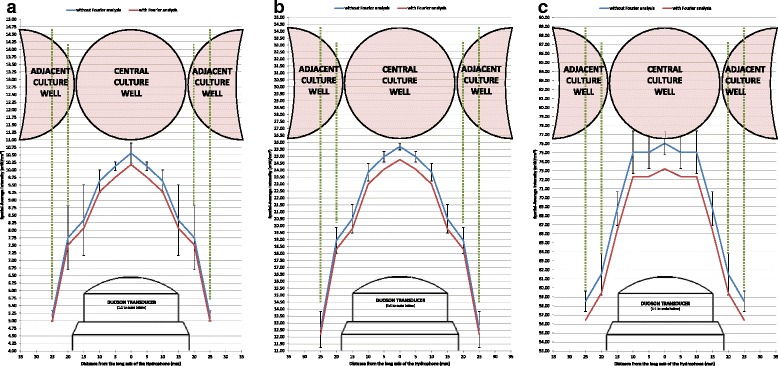
The calculated spatial-average intensity from ultrasound produced from the DuoSon. Spatial-average intensity calculated when a 10 **a** **,** 25 **b**, and 75 mW/cm^2^ **c** ultrasound beam is produced from the DuoSon transducer. Dimensions of the transducer and culture wells are to a 1:1 scale with the horizontal axis. A diagrammatic representation of the culture wells in a six-well plate have been superimposed to demonstrate proximity of the culture wells to each other and their spatial relationship to the ultrasound beam and average intensities. Intensity without Fourier analysis is shown as mean ± SD

Maximum voltage and frequency measurements of ultrasound produced from the DuoSon are shown in Table 1. These values were used to calculate spatial-average intensities as described in the methods section. Beam plots of calculated intensities are shown in Fig. 4a–c. The data indicate that the measurements recorded where the transducer and hydrophone were centrally aligned showed some resemblance to the intensities quoted by the manufacturers. Measurements made horizontally away from the long axis of the transducer showed a gradual reduction of the average intensity. Figure 4 also displays the size of the transducer and positioning of culture wells in a six-well plate which are a 1:1 scale with the horizontal axis. Horizontal measurements show that at 20 and 25 mm from the central axis of the transducer, the calculated intensities without Fourier analysis were 7.75 and 5.2 mW/cm^2^, respectively, when an ultrasound beam using the preset 10 mW/cm^2^ mode is selected. An ultrasound beam produced using the preset 25 mW/cm^2^ mode recorded an average intensity of 19 and 12.5 mW/cm^2^, and when using the 75 mW/cm^2^ mode, 61.5 and 58.5 mW/cm^2^ was recorded at 20 and 25 mm, respectively, from the central axis of the transducer. The beam plots of the 10 and 25 mW/cm^2^ modes (Fig. 4a, b) are similar in form, as opposed to that of the 75 mW/cm^2^ mode (Fig. 4c). The 75 mW/cm^2^ mode produces an ultrasound beam which has a flatter peak. These data imply that when biological cells cultured in dishes of a six-well plate are treated with ultrasound, adjacent culture wells will also be exposed to an ultrasound field.

**Table 1:** The recorded maximum voltage and frequency of ultrasound produced from the DuoSon. Recorded maximum voltage and frequency when a 10, 25, and 75 mW/cm^2^ ultrasound beam is produced from the DuoSon transducer. Measurements were taken at 1, 2, 3, 4, 5, 6, 7, 8, 9, and 10 mm vertically from the transducer face at 0, 5, 10, 15, 20, and 25 mm horizontally from the long axis of the transducer

DuoSon preset intensity (mW/cm^2^)	Vertical distance from transducer face (mm)	Horizontal distance from the long axis of the transducer (mm)	Maximum voltage (mV)	Frequency (kHz)
10	1	0	2.43	47.55
10	2	0	2.41	47.57
10	3	0	2.43	47.55
10	4	0	2.38	47.53
10	5	0	2.39	47.52
10	6	0	2.49	47.53
10	7	0	2.36	47.5
10	8	0	2.39	47.47
10	9	0	2.36	47.46
10	10	0	2.38	47.43
10	1	5	2.38	47.58
10	2	5	2.37	47.57
10	3	5	2.36	47.56
10	4	5	2.36	47.55
10	5	5	2.36	47.54
10	6	5	2.36	47.53
10	7	5	2.36	47.52
10	8	5	2.34	47.5
10	9	5	2.32	47.48
10	10	5	2.32	47.47
10	1	10	2.36	47.54
10	2	10	2.36	47.53
10	3	10	2.33	47.52
10	4	10	2.3	47.53
10	5	10	2.29	47.53
10	6	10	2.28	47.5
10	7	10	2.27	47.45
10	8	10	2.26	47.44
10	9	10	2.25	47.43
10	10	10	2.23	47.43
10	1	15	1.93	46.74
10	2	15	1.92	46.89
10	3	15	1.91	47.08
10	4	15	1.99	47.35
10	5	15	2.14	47.45
10	6	15	2.19	47.46
10	7	15	2.26	47.46
10	8	15	2.27	47.43
10	9	15	2.31	47.41
10	10	15	2.25	47.41
10	1	20	2.09	47.36
10	2	20	2.17	47.38
10	3	20	2.22	47.37
10	4	20	2.21	47.36
10	5	20	2.1	47.36
10	6	20	2.06	47.35
10	7	20	2.01	47.34
10	8	20	1.95	47.33
10	9	20	1.84	47.3
10	10	20	1.81	47.31
10	1	25	1.6	47.2
10	2	25	1.68	47.22
10	3	25	1.7	47.24
10	4	25	1.69	47.26
10	5	25	1.66	47.29
10	6	25	1.69	47.31
10	7	25	1.69	47.3
10	8	25	1.66	47.3
10	9	25	1.65	47.29
10	10	25	1.66	47.32
25	1	0	3.75	47.4
25	2	0	3.76	47.43
25	3	0	3.74	47.43
25	4	0	3.76	47.42
25	5	0	3.75	47.43
25	6	0	3.74	47.42
25	7	0	3.74	47.41
25	8	0	3.72	47.41
25	9	0	3.72	47.4
25	10	0	3.7	47.41
25	1	5	3.71	47.45
25	2	5	3.7	47.43
25	3	5	3.7	47.43
25	4	5	3.71	47.44
25	5	5	3.71	47.43
25	6	5	3.7	47.42
25	7	5	3.68	47.4
25	8	5	3.64	47.37
25	9	5	3.64	47.36
25	10	5	3.63	47.35
25	1	10	3.61	47.43
25	2	10	3.69	47.42
25	3	10	3.65	47.42
25	4	10	3.63	47.41
25	5	10	3.61	47.4
25	6	10	3.59	47.4
25	7	10	3.57	47.38
25	8	10	3.57	47.36
25	9	10	3.54	47.35
25	10	10	3.52	47.34
25	1	15	3.26	46.82
25	2	15	3.17	46.89
25	3	15	3.21	46.95
25	4	15	3.24	47.3
25	5	15	3.3	47.43
25	6	15	3.36	47.42
25	7	15	3.38	47.42
25	8	15	3.47	47.35
25	9	15	3.42	47.34
25	10	15	3.39	47.31
25	1	20	3.02	47.16
25	2	20	3.16	47.36
25	3	20	3.17	47.37
25	4	20	3.2	47.41
25	5	20	3.26	47.42
25	6	20	3.29	47.41
25	7	20	3.29	47.42
25	8	20	3.3	47.43
25	9	20	3.22	47.43
25	10	20	3.15	47.44
25	1	25	2.54	47.32
25	2	25	2.48	47.31
25	3	25	2.65	47.31
25	4	25	2.69	47.3
25	5	25	2.73	47.29
25	6	25	2.77	47.3
25	7	25	2.73	47.3
25	8	25	2.58	47.29
25	9	25	2.45	47.28
25	10	25	2.37	47.27
75	1	0	6.56	47.52
75	2	0	6.5	47.51
75	3	0	6.38	47.52
75	4	0	6.4	47.52
75	5	0	6.43	47.52
75	6	0	6.45	47.51
75	7	0	6.46	47.5
75	8	0	6.47	47.5
75	9	0	6.41	47.49
75	10	0	6.41	47.47
75	1	5	6.5	47.49
75	2	5	6.46	47.5
75	3	5	6.45	47.5
75	4	5	6.43	47.51
75	5	5	6.43	47.52
75	6	5	6.43	47.52
75	7	5	6.44	47.53
75	8	5	6.35	47.51
75	9	5	6.29	47.5
75	10	5	6.26	47.5
75	1	10	6.48	47.46
75	2	10	6.49	47.47
75	3	10	6.51	47.48
75	4	10	6.49	47.49
75	5	10	6.44	47.5
75	6	10	6.42	47.5
75	7	10	6.37	47.5
75	8	10	6.31	47.54
75	9	10	6.27	47.51
75	10	10	6.24	47.5
75	1	15	6.04	47.44
75	2	15	6.07	47.44
75	3	15	6.07	47.47
75	4	15	6.09	47.49
75	5	15	6.15	47.49
75	6	15	6.24	47.52
75	7	15	6.23	47.51
75	8	15	6.22	47.53
75	9	15	6.2	47.55
75	10	15	5.99	47.53
75	1	20	5.75	47.32
75	2	20	5.79	47.39
75	3	20	5.8	47.42
75	4	20	5.83	47.42
75	5	20	5.86	47.45
75	6	20	5.88	47.47
75	7	20	5.89	47.48
75	8	20	5.88	47.5
75	9	20	5.62	47.5
75	10	20	5.59	47.52
75	1	25	5.62	47.3
75	2	25	5.66	47.31
75	3	25	5.64	47.32
75	4	25	5.66	47.38
75	5	25	5.71	47.41
75	6	25	5.65	47.41
75	7	25	5.69	47.42
75	8	25	5.58	47.46
75	9	25	5.61	47.49
75	10	25	5.56	47.53

### Temperature and apparatus

**Fig. 5:**
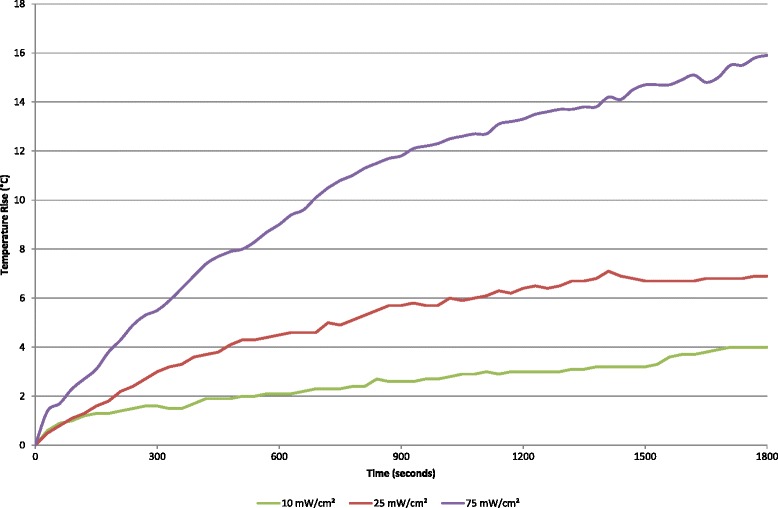
Temperature changes with ultrasound treatment. Temperature changes in culture medium in a well of the six-well plate which is directly exposed with 45-kHz ultrasound over a 30-min period

Temperature measurements indicated that ultrasound with a frequency of 45 kHz, and at the three specified intensities, did not significantly affect the temperature of the culture medium in culture wells adjacent to and distant from the well being treated with ultrasound. Measurements also confirmed that the water bath setup was able to keep the temperature of the culture medium stable at 37 °C (±1 °C). Figure 5 shows the temperature rise in the culture medium of the culture well with the transducer submerged and producing ultrasound. The highest of the three intensities, 75 mW/cm^2^, produced a temperature rise of nearly 16 °C after 30 min of ultrasound exposure. Intensities of 10 and 25 mW/cm^2^ increased the temperature of the medium resulting in maximum temperatures of 4 and 7 °C, respectively, over 30 min of ultrasound exposure. It was observed for the lower two intensities, the temperature rise reached a plateau before the maximum treatment time of the device was reached. This did not occur at the highest intensity. After 5 min (300 sec) of ultrasound treatment, the temperature of the culture medium had risen by 1.6, 3, and 5.5 °C with intensities, 10, 25, and 75 mW/cm^2^, respectively. These data indicate that treatment with ultrasound of a short duration using this method only marginally increases the ambient temperature of the culture medium, but longer times up to 30 min can generate a significant temperature rise.

### Cell number and viability

**Fig. 6:**
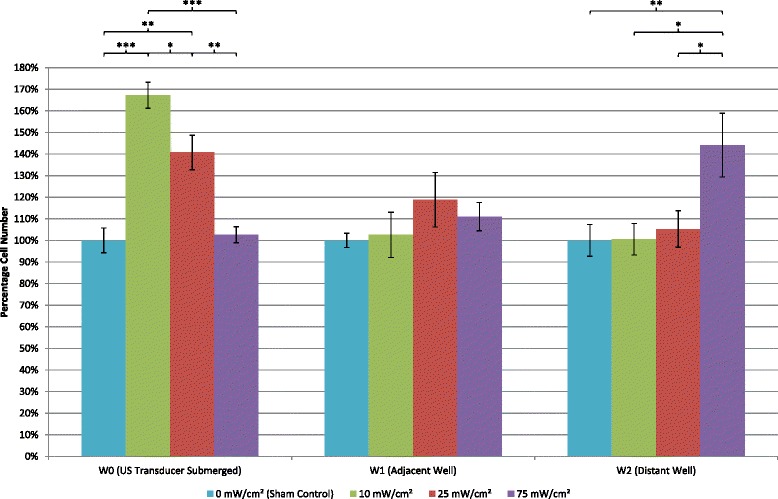
Effects of 45-kHz ultrasound on MDPC-23 cell proliferation. Cell numbers were determined after 8 days of culture in six-well plates with alternating days of ultrasound treatment. Three groups had ultrasound treatment each with the intensities, 10, 25, and 75 mW/cm^2^. A sham-treatment control group had no ultrasound applied to the cells. Total viable cell number is shown for each culture well (W0, W1, and W2), and data is expressed as a percentage of the sham-control group (mean ± SD; *n* = 3). One way ANOVA statistical analysis was carried out, and the statistical significance is indicated (*** *p* < 0.001; ** *p* < 0.01; * *p* < 0.05)

**Fig. 7:**
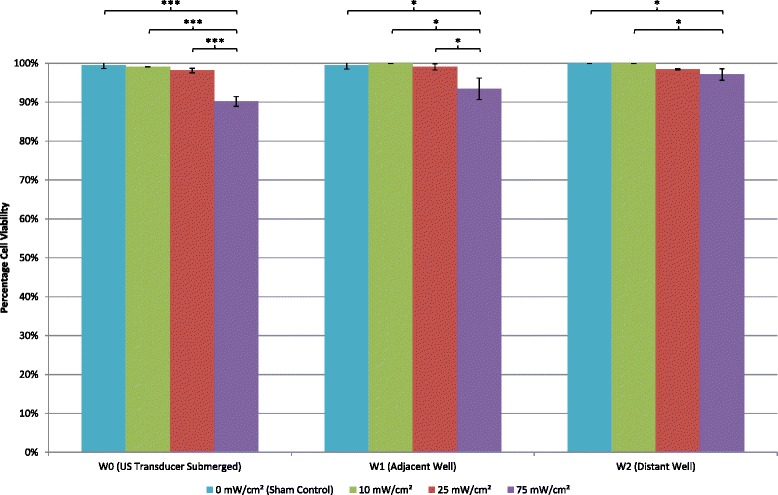
Effects of 45-kHz ultrasound on cell viability of MDPC-23 cells. Viability was determined after 8 days of culture in six-well plates with alternating days of ultrasound treatment. Three groups had ultrasound treatment each with the intensities, 10, 25, and 75 mW/cm^2^. A sham-treatment control group had no ultrasound applied to the cells. Cell viability is shown for each culture well (W0, W1, and W2), and data is expressed as a percentage of the total cell number (mean ± SD; *n* = 3). One way ANOVA statistical analysis was carried out, and the statistical significance is indicated (*** *p* < 0.001; ** *p* < 0.01; * *p* < 0.05)

Application of a 45-kHz ultrasound at the two preset lower intensity levels of 10 and 25 mW/cm^2^ resulted in cell counts from the directly exposed W0 culture well to be significantly higher than the sham-treated group (*p* < 0.001 and *p* < 0.01 respectively) indicating ultrasound-stimulated cell proliferation. The highest preset intensity level, 75 mW/cm^2^, did not result in a significant difference in cell number (Fig. 6), compared to sham; however, cell viability was reduced to 90 % as shown in Fig. 7. The lower intensity levels of 10 and 25 mW/cm^2^ reported higher cell viabilities of 98 % and above (Fig. 7). This indicates that higher ultrasound intensities are not as well tolerated by MDPC-23 cells compared to the lower intensities used in this study. This result is statistically significant (*p* < 0.001).

No significant findings were reported from cell counts from the immediately adjacent W1 culture wells, which were not directly exposed, although cell numbers were marginally increased by approximately 20 and 10 % with the two preset intensity levels of 25 and 75 mW/cm^2^, respectively, compared to the sham control (Fig. 6). Figure 7 shows that cell viability was only marginally and not significantly reduced in adjacent, W1, culture wells when the two lower ultrasound intensities were used; however, with the higher intensity (75 mW/cm^2^), cell viability was reduced to 93 %. This demonstrates that the higher intensity ultrasound had an effect on the cell viability of MDPC-23 cells cultured in adjacent culture wells while having no effect in the directly exposed wells confirming the dose-dependent nature of the ultrasound effects. This result is statistically significant (*p* < 0.05) when compared to the lower intensities and sham control.

Cell numbers in the distant culture well, W2, were found to be significantly (*p* < 0.01) increased when 75 mW/cm^2^ intensity ultrasound was used compared to the sham control (Fig. 6). However, the lower intensities did not significantly increase cell numbers in the distant culture well as shown in Fig. 6. Figure 7 shows that cell viability was reduced across all three intensities. The most significant reduction, compared to sham, was at the highest intensity, 75 mW/cm^2^, resulting in a cell viability of 97 % (*p* < 0.05). The increase in cell numbers together with a slight decrease in cell viability of MDPC-23 cells cultured in distant wells of six-well plates where 75 mW/cm^2^ intensity ultrasound is used indicates that ultrasound at this intensity has a positive effect on cell number when the cells are not directly exposed. This suggests that there is potential for ultrasound with higher intensities to affect other culture wells in the same multiwell plate, with lower intensities, this effect is not significant.

## Discussion

Kilohertz ultrasound has been advocated as a potential treatment modality for tooth repair [24]. To understand, and ultimately improve the effectiveness of this treatment, it is important to determine how ultrasound stimulates the repair processes within a tooth. Previously, our studies established that low-frequency ultrasound effectively penetrates through tooth tissue layers, and the energy is retained within the central chamber of the tooth [26]. Cells responsible for dentine repair are located at the dentine-pulp interface and stimulation at this site may enhance repair processes to maintain tooth vitality. When undertaking *in vitro* experiments, it is critical to ensure that parameters of the treatment modality and experimental setup are well characterized and controlled. An *in vitro* experiment setup using multiwell culture plates with ultrasound treatment is widely used in the literature allowing direct biological effects of ultrasound on replicate cell cultures to be analyzed. [3, 4, 8, 9, 21, 26, 35–37]. This study measured the propagation and intensity of an ultrasound field with a frequency of 45 kHz. We postulated that ultrasound with this frequency would generate a wide beam profile and when used with multiwell plates, could affect cells cultured in adjacent and distant wells of the same culture plate where ultrasound is applied. Figure 4 demonstrates that the 45-kHz ultrasound beam profile had a significant lateral spread potentially crossing over to adjacent non-exposed culture wells. At 25 mm from the central axis of the DuoSon transducer, ultrasound at intensities of 51, 50, and 78 % of manufacturers preset intensities were found (10, 25, and 75 mW/cm^2^, respectively). Due to apparatus limitations, measurements beyond this point could not be made; however, the data and theoretical knowledge of ultrasound propagation suggests that there could be further lateral propagation. It is important to consider that ultrasound-characterization measurements reported in this study are in “free-field” conditions and different to the experiment apparatus and culture multiwell setup. Culture wells shown in Fig. 4 are superimposed to scale to demonstrate proximity. However, this study provides biological evidence to support ultrasound propagation in this way by considering the findings of cell number and viability in W1 and W2 culture wells of the six-well plate (Figs. 6 and 7). The findings have a major influence on future *in vitro* cell-culture study designs where ultrasound is applied to multiwell culture plates. Figure 4 shows the intensity measured and calculated directly over the central axis of the transducer. This can be considered the central or core intensity of the ultrasound beam and is frequently the intensity quoted by manufacturers in their documentation or displayed on the device when in use. To ensure robustness of the data collected, ultrasonic output must be characterized prior to use for *in vitro* study [38]. In this study, the manufacturer’s quoted intensities aligned well with the intensities calculated without Fourier analysis (Fig. 4); however, the intensities calculated with Fourier analysis were lower. This may partially be due to the fact that the Fourier-transform calculation determines the intensity over multiple frequencies. Furthermore, as the hydrophone sensitivity is rarely constant as a function of frequency, interpolation was used to determine the correct calibration factor which caused the marginal discrepancy seen between calculated intensities with and without Fourier analysis. Another source for this discrepancy could be due to the use of null values when completing the data set in order to obtain 2^*n*^ (*n* = 1, 2, …) data points which could introduce marginal errors in the Fourier coefficients, which in turn may have hindered the outcome of the original signal.

Thermal variation in cell culture medium with ultrasound treatment is a concern since a temperature rise greater than 5 °C above 37 °C could adversely affect cell viability [39]. In this study, 45-kHz ultrasound with an intensity of 75 mW/cm^2^ registered a temperature rise of slightly over 5 °C during the 5-min treatment time resulting in reduced cell viability. This was found in culture well, W0, which was directly exposed to ultrasound (Fig. 5). No thermal variation was found in adjacent (W1) and distant (W2) culture wells during ultrasound treatment. A larger volume of culture medium (9 ml), than usually used, was required in each W0 culture well to ensure the radiating surface of the ultrasound transducer could be submerged into the culture medium but also allow space for any potential heat generated by the ultrasound transducer to be dissipated (Figs. 2 and 3). Even with this precaution, the setup described should only be used to treat cells directly with ultrasound for 5 min per episode of treatment with the highest of the three intensities. At the two lower intensities, a single treatment episode can be delivered for up to 10 min with 25 mW/cm^2^ before a temperature rise of 5 °C is registered and the 10 mW/cm^2^ plateaus at 4 °C. Temperature changes in apparatus setup have also been investigated by Leskinen [40]. They confirmed that temperature variation reflects biological outcome and advocate detailed temperature characterization with *in vitro* ultrasound exposures. Our results show that cell viability was significantly (albeit moderately) reduced in all three culture-well groups when ultrasound with the highest intensity was employed. Temperature variation in the directly exposed, W0, culture well could potentially account for the reduced cell viability in this well; however, there was no thermal variation in culture wells W1 and W2. The same intensity level also significantly increased cell numbers in the distant W2 culture well (Fig. 6) without a change in temperature. Although many studies have reported therapeutic biological effects [8, 9, 23, 24, 26, 33, 41–45], it is not fully understood how ultrasound triggers a response in cells and tissues. Two broad categories, thermal and non-thermal, have been postulated and discussed by researchers [28, 32], and it is thought by some that it may only be a thermal effect that brings about changes in biological tissues. This study indicates that non-thermal biomechanical effects should be considered as a temperature rise was not recorded in culture wells W1 and W2, but significant changes to cell number and viability were found. Similarly, Fig. 5 shows that the lowest intensity setting recorded a marginal temperature rise of less than 2 °C during the 5-min treatment time. This resulted in the highest increase in cell number compared to sham (Fig. 6). Thus, it can be postulated that the mechanism of action in this case is mechanical stimulation possibly via microstreaming effects on the cell membrane transmitted through the cytoskeleton and ultimately leading to increased mitosis. Data collected in this study also indicates that higher intensities may prove too large a stimulus for the cell and result in irreversible cell damage and death. Further studies are required to identify specifically how a cell is stimulated by ultrasound to produce a response.

When considering the apparatus and logistics of treating biological cells with ultrasound, an increase in temperature can also have an effect on the ultrasonic output of the transducer [46]. Materials used in the construction of ultrasonic transducers are thermally sensitive, and temperatures outside the materials working parameters affect the intensity of ultrasound produced. Standing waves are a concern when the ultrasound beam meets a surface which is perpendicular to its direction of travel [47]. In the setup described, the effect of standing waves cannot be excluded as the transducer is at right angles to the culture surface of the six-well plate. To reduce standing waves, the direction of the ultrasound beam can be angled to prevent such reflections occurring, or the transducer can be kept in motion during ultrasound treatment. The latter solution is also useful to prevent the build-up of heat; however, the movement of either the transducer or the culture plate may result in a reduction of cell viability. These factors make it important to characterize the ultrasonic output using the same conditions and equipment as when cells are treated with ultrasound; however, this can be extremely difficult and in some cases nearly impossible when working with *in vitro* cell culture.

## Conclusions

This study showed that low-frequency ultrasound has a beam profile with significant lateral spread reaching and affecting cell cultures in adjacent wells of a multiwell culture plate. Cells from culture wells directly exposed to ultrasound demonstrated both a change in temperature and a biological affect. This is in contrast to findings from culture wells not directly treated with ultrasound where a biological effect was reported without a temperature rise. This adds to the evidence of a mechanical effect of ultrasound on biological cells. This study demonstrates the importance of characterizing the ultrasonic output from equipment and questions the suitability of multiwell culture plates for low-frequency ultrasound application.
